# Effects of leptin on sympathetic nerve activity in conscious mice

**DOI:** 10.14814/phy2.12554

**Published:** 2015-09-17

**Authors:** Donald A Morgan, Fabien Despas, Kamal Rahmouni

**Affiliations:** 1 Department of Pharmacology, University of Iowa Carver College of MedicineIowa City, Iowa; 2 Department of Internal Medicine, University of Iowa Carver College of MedicineIowa City, Iowa

**Keywords:** Leptin, sodium nitroprusside, renal, lumbar, mice, sympathetic nerve activity

## Abstract

The adipocyte-derived hormone, leptin, has emerged as an important regulator of regional sympathetic nerve activity (SNA) with pathophysiological implications in obesity. Genetically engineered mice are useful to understand the molecular pathways underlying the SNA responses evoked by leptin. However, so far the effect of leptin on direct SNA in mice has been studied under general anesthesia. Here, we examined the sympathetic responses evoked by leptin in conscious mice. Mice were instrumented, under ketamine/xylazine anesthesia, with renal or lumbar SNA recordings using a thin (40 gauge) bipolar platinum–iridium wire. The electrodes were exteriorized at the nape of the neck and mice were allowed (5 h) to recover from anesthesia. Interestingly, the reflex increases in renal and lumbar SNA caused by sodium nitroprusside (SNP)-induced hypotension was higher in the conscious phase versus the anesthetized state, whereas the increase in both renal and lumbar SNA evoked by leptin did not differ between anesthetized or conscious mice. Next, we assessed whether isoflurane anesthesia would yield a better outcome. Again, the SNP-induced increase in renal SNA and baroreceptor-renal SNA reflex were significantly elevated in the conscious states relative to isoflurane-anesthetized phase, but the renal SNA response induced by leptin in the conscious states were qualitatively comparable to those evoked above. Thus, despite improvement in sympathetic reflexes in conscious mice the sympathetic responses evoked by leptin mimic those induced during anesthesia.

## Introduction

The sympathetic nervous system is a major efferent regulatory mechanism that links the central nervous system with visceral effectors to control multiple physiological functions including vascular resistance, heart rate, blood pressure, renal function, thermoregulation, and metabolism (Webber and Macdonald 2000; Dulloo 2002; Malpas 2010). Data from a range of experimental paradigms support the significance of the sympathetic nervous system for homeostasis and the coordination of physiological responses such as the cardiovascular and metabolic events in the case of fasting or exposure to cold (Dulloo 2002; Landsberg 2006). In addition, alterations in sympathetic nerve activity (SNA) have been implicated in the development and progression of several diseases. For instance, it is now well recognized that excessive sympathetic traffic is a major factor in causing hypertension and other cardiovascular diseases in humans and in animal models (Osborn et al. 1997; Esler 2011).

Although a number of hemodynamic, pharmacological, and biochemical techniques have been developed and used to evaluate sympathetic tone, direct recording from the nerve fibers is considered one of the most accurate ways to study the activity of the sympathetic nervous system (Grassi and Esler 1999; Malpas 2010; Stocker and Muntzel 2013). In addition to the possibility of measuring the nerve activity (e.g., counting the number of spikes and integrated voltage) over a period of time, one major advantage of this technique is that it can be used to simultaneously assess SNA subserving different tissues/beds with other physiological parameters such as blood pressure and heart rate (Rahmouni and Morgan 2007; Morgan and Rahmouni 2010). Direct SNA recording has been used extensively to study sympathetic regulation in humans and animal models (Grassi 2009; Burke et al. 2011).

Leptin is an adipocyte-derived hormone which acts in the central nervous system to decrease food intake and increase energy expenditure by activating thermogenic SNA (Elmquist et al. 1999; Morton et al. 2006). Leptin also increases SNA to organs involved in cardiovascular regulation such as the kidney and induces arterial pressure elevation (Rahmouni 2010). In addition, the sympathoexcitatory effect of leptin has emerged as an important culprit linking obesity and hypertension (Rahmouni et al. 2005a; Hall et al. 2010; Jeppesen and Asferg 2010; Rahmouni 2014). This highlights the importance of understanding the mechanisms involved in leptin regulation of SNA.

The availability of genetically engineered mice has provided a powerful tool to investigate the molecular pathways underlying leptin effects on SNA. However, so far the effect of leptin on direct SNA in mice has been studied under general anesthesia. Thus, in the present study we examined whether the sympathetic responses evoked by leptin are different in conscious mice. For this, we systematically compared baseline SNA, reflex responses to decreases in arterial pressure, and responses to the sympathoexcitatory effects of leptin in conscious versus anesthetized states in mice.

## Material and Methods

### Animals

All studies were approved by The University of Iowa Animal Research Committee and conducted in accordance with the National Institutes of Health Guide for the Care and Use of Laboratory Animals. Male wild-type mice from our own colony were housed in groups of 3–5 at 23°C with a 12-h light/dark cycle and ad libitum access to normal chow diet and tap water.

### General surgical procedures

Anesthesia was induced using intraperitoneal ketamine (91 mg/kg) and xylazine (9.1 mg/kg) or isoflurane (up to 5% for induction; 1.5–2% for maintenance). The right jugular vein was cannulated with microrenathane tubing (MRE-40). In ketamine/xylazine-anesthetized mice the tubing inserted in the jugular vein was filled with diluted ketamine (1.13 mg/mL)/xylazine (0.11 mg/mL) solution used to sustain the level of anesthesia throughout the entire surgical procedure. In other cohorts of mice (leptin treatment protocol), anesthesia induced by ketamine/xylazine was maintained with *α*-chloralose (initial dose of 25 mg/kg and sustaining dose of 6 mg/kg per h) as described previously (Morgan et al. 2008; Harlan et al. 2011). Next, the left carotid artery was cannulated with microrenathane tubing (MRE-40) for continuous arterial pressure measurement.

The right jugular venous catheter was tunneled subcutaneously behind the left forelimb. Likewise, the left carotid artery catheter was tunneled behind the right forelimb where both arterial and venous catheters were exited at the nape of the neck. A 6-0 vicryl suture was used to secure the catheters to the muscle layer under the skin and to close the incision in the neck region.

### Sympathetic nerve recordings

**Figure 1:**
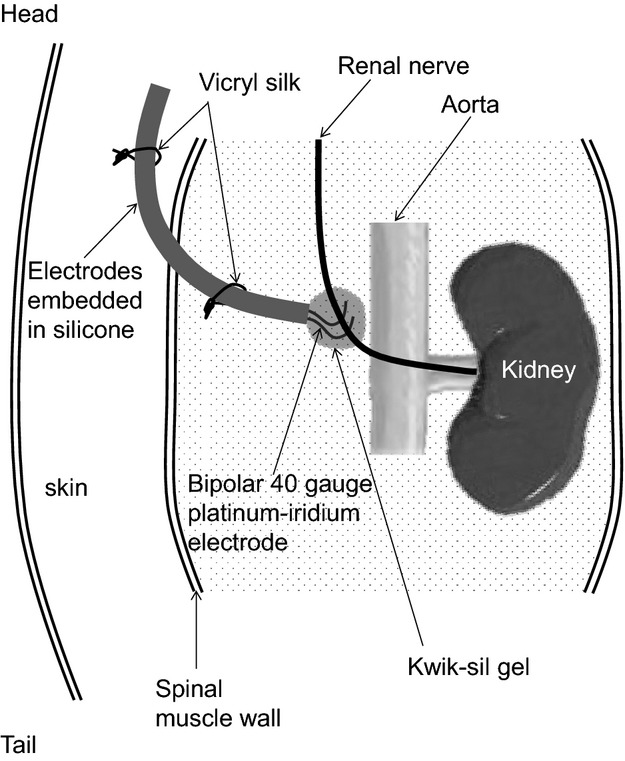
Schematic illustration of the method for implanting electrodes for conscious renal SNA recording in mice. Note the head/tail orientation of the mouse. See text in the Material and Methods section for detailed explanation.

Sympathetic nerve activity was measured by multifiber recording directly from the nerves subserving the kidney or hindlimb. The left kidney or lumbar sympathetic nerves were exposed through a left retroperitoneal incision. Magnification of the surgical field with a dissecting microscope allowed a careful dissection of a nerve branch with minimum disruption of the tissues and blood supply surrounding the nerve. Each nerve was then placed on a thin (40 gauge) bipolar platinum–iridium electrode (Cooner Wire, Chatsworth, CA). After an optimal positioning of the electrode (based on signal-to-noise ratio) was obtained, the electrode was encased with a minimal amount (˜100 *μ*L) of silicone gel (Kwik-Cast; WPI, Sarasota, FL) that allowed the movement of the assembly without breaking the nerve (Fig. 1). A separate grounding wire was inserted in the nearby muscle to ground the mouse.

A 6-0 vicryl silk was used to stitch the recording electrodes to the spinal muscle. Vicryl silk was also used to secure the electrodes along the subcutaneous dorsal surface until these electrode wires were exteriorized at the nape of the neck to where the arterial and venous catheters were positioned. Finally, 6-0 silk sutures were used to close the skin incisions.

The nerve electrodes were attached to a high-impedance probe (HIP-511, Grass Instruments Co., Quincy, MA). The signal was amplified 10^5^ times with a Grass P5 AC preamplifier, and band-pass filtered at 100–1000 Hz before it was sent to a speaker system and oscilloscope (model 54501A, Hewlett-Packard Co., Palo Alto, CA). Listening to the audible nature of the sympathetic discharge and the visualization of the signal on the oscilloscope allowed a continuous assessment of the quality of the SNA recording. The nerve traffic analyzer counted the action potentials (spikes/sec) that exceeded the background noise threshold. The original neurogram was also routed to a MacLab analog-digital converter (model 8S, Ad Instruments, Castle Hill, New South Wales, Australia) for a permanent recording and later analysis using a Macintosh computer. The noise level in the recording of SNA was evaluated at the end of each experiment at death.

Once the surgical procedure was completed, each mouse was placed in a plexiglass chamber (3.6 cm inner diameter, 5 cm outer diameter, and 12.5 cm in length which can accommodate a 15- to 30-g mouse with free forward/backward movement, but restricted from turning around) for recovery. Continuous observation was performed until the mouse reached full consciousness. Ketamine/xylaxine-induced anesthesia typically subsided for about 1 h after termination of infusion of anesthetics and reflex responses (such as leg movement or whisker twitches) to tail and foot pad pinches were fully restored after 5 h (referred to as the conscious state). In contrast, mice regain consciousness very quickly after termination of isoflurane and were studied 5 h and 24 h after recovery.

### Experimental protocols

#### Effects of sodium nitroprusside

Sodium nitroprusside (SNP) was used to study the reflex increase in the renal and lumbar SNA during arterial pressure decrease. Shortly after completing the surgical procedures (15–30 min) while the mice are still anesthetized, we measured hemodynamic parameters and SNA, at baseline and during intravenous bolus administration of 5 *μ*g (in 5 *μ*L) of sodium nitroprusside (SNP). Mice were then allowed to recover from the anesthesia (as described above) before the hemodynamic and SNA responses to intravenous administration of SNP (5 *μ*g in 5 *μ*L) were assessed in the conscious state.

#### Sympathetic responses induced by leptin

We tested the sympathetic nerve activation evoked by leptin in conscious mice as compared to anesthetized mice in which anesthesia was maintained with *α*-chloralose (initial dose of 25 mg/kg and sustaining dose of 6 mg/kg per h) as in our previous studies (Morgan et al. 2008; Harlan et al. 2011). Renal and lumbar SNA, measured in separate cohorts of mice, were recorded during a 10-min control period, followed by intravenous administration of leptin (60 *μ*g) or vehicle (saline, 60 *μ*L). Renal and lumbar SNA were recorded for the next 4 h after each treatment.

### Data analysis

Baseline renal and lumbar SNA are expressed in spikes/sec, while the SNA responses are expressed as percent change from baseline with 0% as baseline. All data are expressed as mean ± SEM. Comparisons between anesthetized and conscious states, measured in the same mice, were made using paired Student’s *t*-test. One- or two-way analyses of variance (ANOVAs) with or without repeated measures were used to compare multiple groups and time points followed by a post hoc test when ANOVA reached significance. A *P* < 0.05 was considered statistically significant.

## Results

**Figure 2:**
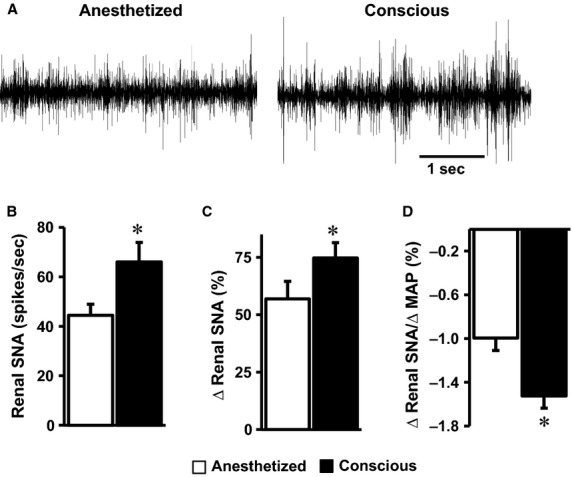
Comparison of baseline renal SNA (A, B) and the effect of intravenous administration of SNP on renal SNA (C) and baroreflex index (D) in the conscious or the anesthetized (with ketamine/xylazine) state in mice. Segments of original records of baseline renal SNA (A) and average data (B) are shown. *P < 0.05 versus anesthetized, *n* = 15 per group.

We compared the baseline hemodynamic and sympathetic parameters between anesthetized state (with ketamine/xylazine) and conscious state (5 h after recovery from anesthesia) in the same mice. We found that mean arterial pressure (MAP) and heart rate (HR) were significantly (*P* < 0.002) higher when the mice are conscious (94 ± 5 mmHg and 437 ± 23 bpm, respectively) as compared to when they were anesthetized (78 ± 5 mmHg and 302 ± 16 bpm, respectively). Baseline renal SNA was also higher in the conscious state relative to the anesthetized state (Fig. 2A and B).

We also quantified the hemodynamic and sympathetic effects induced by SNP between anesthetized and conscious states measured in the same mice. Intravenous administration of SNP caused a substantial and comparable (*P* = 0.33) drop in MAP when the mice were anesthetized (–36 ± 3 mmHg) and conscious (–34 ± 4 mmHg). As expected, SNP-induced hypotension caused a reflex increase in renal SNA. This reflex rise in renal SNA evoked by SNP was significantly higher (*P* = 0.001) when the mice were conscious or anesthetized (Fig. 2C). The increase in renal SNA relative to the decrease in MAP, an index of baroreceptor-renal SNA reflex, was also elevated (*P* = 0.0009) when the mice were conscious or anesthetized (Fig. 2D).

**Figure 3:**
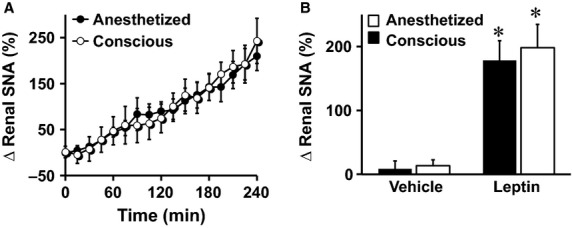
Comparison of renal SNA response to intravenous administration of leptin (60 *μ*g) between anesthetized (induced with ketamine/xylazine and sustained with *α*-chloralose) and conscious mice. Time-course (A) and average of the fourth hour (B) of renal SNA responses are displayed. *P < 0.05 versus vehicle, *n* = 8 (conscious–vehicle), 9 (conscious–leptin), 14 (anesthetized–vehicle), and 15 (anesthetized–leptin).

Next, we compared the sympathoexcitatory effect of leptin between anesthetized and conscious mice. In both anesthetized and conscious mice, intravenous administration of leptin caused a slow, but significant increase in renal SNA (Fig. 3A). Surprisingly, the increase in renal SNA evoked by leptin did not differ between the anesthetized and conscious mice (Fig. 3A and B). Intravenous administration of vehicle had no effect on renal SNA in either anesthetized or conscious mice (Fig. 3B).

**Figure 4:**
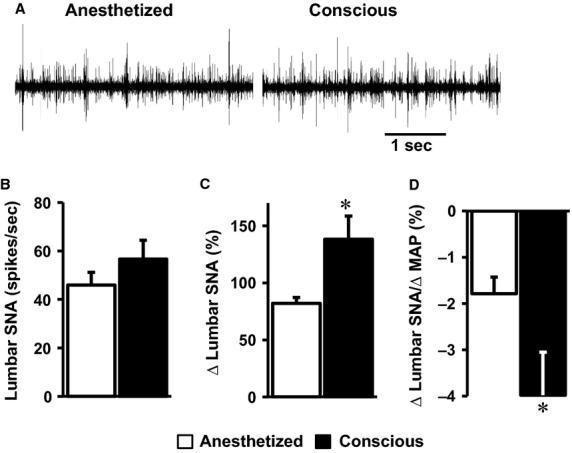
Comparison of baseline lumbar SNA (A, B) and the effect of intravenous administration of SNP on lumbar SNA (B) and baroreflex index (C) in the conscious or the anesthetized (with ketamine/xylazine) state in mice. Segments of original records of baseline lumbar SNA (A) and average data (B) are shown. *P < 0.05 versus anesthetized, *n* = 4 (conscious–vehicle), 5 (conscious–leptin), 12 (anesthetized–vehicle), and 10 (anesthetized–leptin).

Next, we asked if the comparable leptin-induced sympathetic nerve activation between the anesthetized and conscious mice is specific to the renal SNA. In other cohorts of mice, we recorded lumbar SNA in anesthetized versus conscious states. As shown in Figure 4, baseline lumbar SNA tended to be higher in the conscious state relative to the anesthetized state, but this was not statistically different (*P* = 0.16). However, the reflex rise in lumbar SNA evoked by SNP was significantly higher in the conscious state relative to the anesthetized state (*P* = 0.007, Fig. 4C). Moreover, the reflex increase in lumbar SNA relative to the decrease in MAP (sensitivity of the baroreceptor-lumbar SNA reflex) evoked by SNP was elevated (*P* = 0.05) when the mice were conscious versus anesthetized (Fig. 4D).

**Figure 5:**
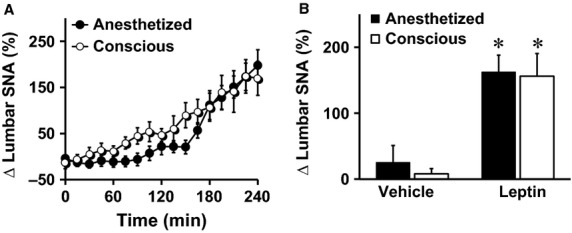
Comparison of lumbar SNA response to intravenous administration of leptin (60 *μ*g) between anesthetized (induced with ketamine/xylazine and sustained with *α*-chloralose) and conscious mice. Time-course (A) and average of the fourth hour (B) of lumbar SNA responses are displayed. *P < 0.05 versus vehicle, *n* = 4–12 per group.

We also compared the lumbar SNA response to leptin between anesthetized and conscious mice. Leptin-induced lumbar sympathetic activation was not statistically different in anesthetized mice when compared to conscious mice (Fig. 5A and B). Vehicle treatment had no significant effect on lumbar SNA in anesthetized or conscious mice (Fig. 5B). Thus, similar to the renal SNA, conscious lumbar SNA recording did not yield a better sympathetic response to leptin.

To test whether the lack of improvement in the SNA responses evoked by leptin is due to the anesthetic or the short-term recovery from the anesthesia/surgery, we performed additional experiments using isoflurane which is well known to have minimal side effects with rapid recovery from anesthesia (Zuurbier et al. 2002, 2014; Szczesny et al. 2004). This made it possible to record SNA after 5 h as well as 24 h with high success rate.

Isoflurane-anesthetized mice had a MAP of 98 ± 3 mmHg and a HR of 519 ± 24 bpm. MAP increased to 111 ± 3 mmHg (*P* < 0.05 vs. anesthetized state) and HR remained unchanged (513 ± 34 bpm) 5 h after recovery from anesthesia. MAP and HR were 105 ± 6 mmHg and 565 ± 32 bpm, respectively, 24 h after recovery from anesthesia.

**Figure 6:**
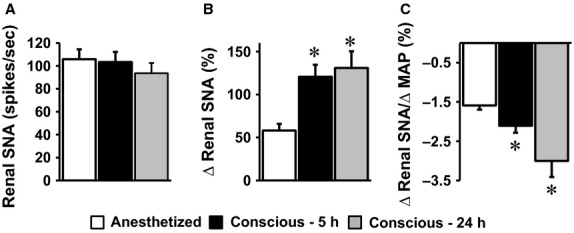
Comparison of baseline renal SNA (A) and the effect of intravenous administration of SNP on renal SNA (B) and baroreflex index (C) in isoflurane-anesthetized state and conscious states (5 and 24 h after anesthesia). *P < 0.05 versus anesthetized, *n* = 14 per group.

Interestingly, renal SNA was not different in isoflurane-anesthetized state compared to conscious state (5 and 24 h after recovery, Fig. 6A). In contrast, SNP-induced increase in renal SNA was significantly elevated in the conscious states relative to the anesthetized state (Fig. 6B). Moreover, the sensitivity of the baroreceptor-renal SNA reflex evoked by SNP was higher in the conscious states versus anesthetized phase with a more pronounced improvement at 24 h versus 5 h post surgery (Fig. 6C).

**Figure 7:**
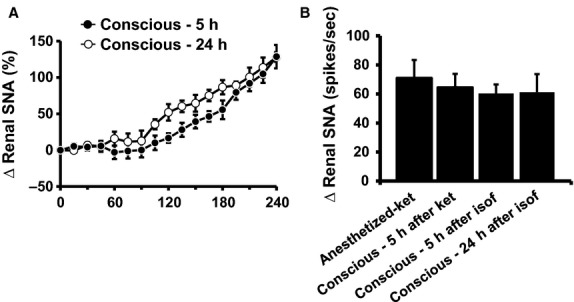
(A) Renal SNA responses to intravenous administration of leptin (60 *μ*g) in conscious mice (5 and 24 h after recovery from isoflurane-anesthesia/surgery). (B) Average of the fourth hour of renal SNA response (expressed in spikes/sec) to leptin under various conditions. *n* = 5–7 per group.

The renal SNA response to leptin was slightly more pronounced, but not significantly different, during the 60- to 180-min period posttreatment at 24 h relative to 5 h postanesthesia/surgery (Fig. 7A). In addition, these responses were qualitatively comparable to those evoked in anesthetized mice or after 5 h recovery from ketamine anesthesia (see Fig. 3), but quantitatively the increase in renal SNA after leptin treatment appears less pronounced in the mice undergoing isoflurane surgery. The difference in baseline renal SNA seems to account for this apparent difference in the intensity of the response because when the increase in renal SNA evoked by leptin was calculated in spikes/sec there was no statistical difference between the various groups (Fig. 7B).

## Discussion

In the present study, we assessed the effect of leptin on sympathetic nerve activity in conscious mice. Careful surgical manipulation associated with some technical modifications was critical to achieve sympathetic nerve recording in conscious mice. In particular, the thinner (40 gauge) electrodes and use of minimal amount of gel to seal the electrode to the nerve made the assembly flexible lowering the risk of damaging the nerve which is the most common issue related to conscious nerve recording. Using this approach, we demonstrate that the baroreceptor-SNA reflex sensitivity was significantly greater when the mice are conscious or anesthetized with ketamine/xylazine or isoflurane. However, the increase in both renal and lumbar SNA evoked by leptin did not differ between anesthetized and conscious mice.

Consistent with our previous findings, leptin caused significant increases in both renal and lumbar SNA in mice (Rahmouni et al. 2005b; Morgan et al. 2008). However, the sympathetic nerve activation induced by leptin was comparable whether the mice were anesthetized or conscious. Moreover, whether ketamine/xylazine or isoflurane were used to sedate the mice did not change significantly the SNA responses evoked by leptin. This result is rather surprising given the improved baroreflex sensitivity which may be expected to yield better sympathetic nerve response to leptin. Nonetheless, the dissociation between baroreflex sensitivity and leptin-induced sympathetic activation is consistent with the lack of effect of leptin treatment on the maximum gain of the renal SNA–MAP curve reported previously in rats (Hausberg et al. 2002). In addition, baroreflex activation was found to suppress renal SNA in rats before and during leptin treatment to a similar extend despite sympathetic activation after leptin (Hausberg et al. 2002).

The significant enhancement in sodium nitroprusside-induced reflex activation of renal and lumbar SNA in the conscious states as compared to the anesthetized state is consistent with the idea that anesthesia has a deleterious effect of on baroreflex sensitivity and sympathetic nerve responses (Smith 2003). Various anesthetic agents were found to alter the sensitivity of baroreflex, sympathetic activity, and hemodynamic parameters in different species ranging from rodents to humans (Shimokawa et al. 1998; Grassi and Esler 1999; Burke et al. 2011). Interestingly, although isoflurane had no effect on baseline renal SNA the sympathetic reflex responses evoked by sodium nitroprusside were attenuated when the mice are under isoflurane anesthesia relative to the conscious phases.

One potential limitation of the current study relates to the fact that SNA responses were assessed in the conscious mice 5–24 h after the anesthesia. However, the relatively greater baseline hemodynamic and sympathetic parameters combined with the improvement in the sodium nitroprusside-induced reflex increase in SNA demonstrates the benefit of measuring SNA in the conscious state even when performed shortly after recovery from anesthesia. Although data are not presented here, we found that measurements of renal SNA can continue 48–72 h after instrumentation in freely moving mice, but with relatively low success rate. Thus, refinement of the technique is allowing longer recordings to increase steadily with the ultimate goal of establishing long-term SNA recording in conscious mice.

With the ever-growing list of genetically engineered mouse models which are now widely used to gain insight into the genetic basis and neural mechanisms that regulate autonomic and sympathetic functions, there is a need for methods that allow accurate assessment of sympathetic phenotypes in mice. The establishment of direct sympathetic nerve recording in mice is relatively recent (Ling et al. 1998; Correia et al. 2002; Ma et al. 2004). The recent report (Hamza and Hall 2012) combined with the present study demonstrating the feasibility of direct sympathetic nerve recording in conscious mice is an important step forward to eliminate the well-established confounding effects of anesthesia. This may also encourage the development of miniature implantable radiotelemetric chronic SNA recording devices that are currently used in rats and other larger animals for application in mice.
